# Wound Healing In Surgery for Trauma (WHIST): statistical analysis plan for a randomised controlled trial comparing standard wound management with negative pressure wound therapy

**DOI:** 10.1186/s13063-019-3282-y

**Published:** 2019-03-28

**Authors:** Ruth Knight, Louise M. Spoors, Matthew L. Costa, Susan J. Dutton

**Affiliations:** 1 Oxford Clinical Trials Research Unit, Centre for Statistics in Medicine, Nuffield Department of Orthopaedics, Rheumatology and Musculoskeletal Sciences, University of Oxford, Windmill Road, Oxford, OX3 7LD UK; 2 Oxford Trauma, Nuffield Department of Orthopaedics, Rheumatology and Musculoskeletal Sciences, University of Oxford, Oxford, UK

**Keywords:** Statistical analysis plan, Randomised controlled trial, Negative pressure wound therapy, Lower extremity trauma, Surgical site infection

## Abstract

**Background:**

In the context of major trauma, the rate of wound infection in surgical incisions created during fracture fixation amongst patients with closed high-energy injuries is high. One of the factors which may reduce the risk of surgical site infection is the type of dressing applied over the closed incision. The WHIST trial evaluates the effects of negative-pressure wound therapy (NPWT) compared with standard dressings.

**Methods/design:**

The WHIST trial is a multicentre, parallel group, randomised controlled trial. The primary outcome is the rate of deep surgical site infection at 30 days after major trauma. Secondary outcomes are measured at 3 and 6 months post-randomisation and include the Disability Rating Index, the EuroQoL EQ-5D-5 L, the Doleur Neuropathique Questionnaire, a patient-reported scar assessment, and record of complications. The analysis approaches for the primary and secondary outcomes are described here, as are the descriptive statistics which will be reported. The full WHIST protocol has already been published.

**Discussion:**

This paper provides details of the planned statistical analyses for this trial and will reduce the risks of outcome reporting bias and data driven results.

**Trial registration:**

International Standard Randomised Controlled Trials database, ISRCTN12702354. Registered on 9 December 2015.

## Background

Major trauma is the leading cause of death in patients under 45 years and a significant cause of short- and long-term morbidity [1]. In the context of major trauma, the wounds associated with surgery to fractured limbs are notoriously difficult to manage. Even in closed high-energy injuries associated with major trauma, the rate of infection in surgical incisions created during fracture fixation remains high; tibial plateau fractures are associated with infection rates of up to 27% [2–6] while pilon fractures have an incidence of deep infection ranging from 5 to 40% [7–10]. If surgical site infection does occur, treatment frequently continues for years after the trauma with significant personal and societal costs [11].

One of the factors which may reduce the risk of surgical site infection in the surgical wounds of major trauma patients is the type of dressing applied over the closed incision at the completion of the operative procedure. Traditionally, the surgical incision is covered with an adhesive dressing or gauze maintained in place with a bandage to protect the wound from contamination from the outside environment. Negative-pressure wound therapy (NPWT) is an alternative form of dressing which may be applied to closed surgical incisions. In this treatment, an open-cell, solid foam overlies the incision and is covered with a semipermeable membrane. A sealed tube is used to connect the foam to a pump which creates a partial vacuum over the wound.

There has only been one randomised trial comparing standard wound dressing with NPWT for patients with closed surgical wounds following major trauma to the limbs [12]. This trial demonstrated a reduction in the rate of late/deep wound infections in patients treated with NPWT (9%) versus those treated with standard dressings (15%); however, the reduction was of borderline statistical significance (*p* = 0.049), and the study has since been criticised for methodological flaws [13]. In addition, a recent Cochrane review concluded that further trials regarding the effects of NPWT are required [13].

The WHIST trial is a large-scale, multicentre, parallel group, randomised controlled trial designed to compare the rates of deep infection in patients with major trauma requiring surgical incisions for the treatment of lower limb fractures treated with NPWT compared to those treated with standard wound dressings. The protocol paper for the WHIST trial has been published previously [14]; the aim of this paper is to report in detail the analysis plan as agreed by the trial steering committee in March 2018. This paper has been prepared according to the published guidelines on the content of statistical analysis plans [15].

## Methods and design

### Trial design

WHIST is a multicentre, two-arm, parallel-group, superiority randomised controlled trial designed to compare the rates of ‘deep infection’ in patients allocated to standard wound dressing versus those allocated to NPWT. Eligible patients are randomised on a 1:1 basis using minimisation to balance the two treatment groups by trial centre, open or closed fracture at presentation, and Injury Severity Score (ISS) ≤ 15 versus ISS ≥ 16. Neither participants nor their treating surgeons are blind to treatment allocation since wound dressings are clearly visible. The primary outcome is assessed at 30 days after randomisation with secondary outcomes assessed at baseline, 30 days, and 3 and 6 months after randomisation. Full details of the trial design, study population, and study procedures have been published previously [14].

The trial is registered with the International Standard Randomised Controlled Trials database, ISRCTN reference number ISRCTN12702354.

### Objectives

The primary objective of this trial is to quantify and draw inferences on differences in the rate of deep infection of the lower limb in the 30 days after major trauma between participants receiving standard dressings and those receiving NPWT. Secondary objectives include assessing differences between the same groups in disability, wound healing, quality of life, neuropathic pain, and the number and nature of complications experienced at 3 and 6 months post-randomisation.

### Outcomes

#### Primary outcome

The primary outcome for this study is the rate of deep infection; the Centre for Disease Control and Prevention (CDC) definition of a “deep surgical site infection”—a wound infection involving tissues deep to the skin that occurs within 30 days of injury [16]—is used. Since the trial began, the CDC definition of a deep surgical site infection has been modified to include wound infections occurring up to 90 days after injury if metal implants are used in the fracture fixation [17]. Although not all wounds associated with lower limb fracture surgery contain implants, this is the most common method of fixation and therefore we will incorporate this alternative definition of the primary outcome in a supplementary analysis to ensure the study can be utilised in future systematic reviews and meta-analyses.

#### Secondary outcomes

The secondary outcome measures, recorded at 3 and 6 months post-injury unless otherwise stated, are as follows:

- Disability Rating Index (DRI) [18]: a self-administered, 12-item visual analogue scale (VAS). Each item is scored from 0 (carry out task without difficulty) to 100 (not at all). Total scores are calculated as an average across all 12 items with higher scores indicating greater disability.
- Euroqol EQ-5D-5 L [19, 20]: a self-reported outcome measure consisting of five dimensions each with five possible responses which are converted to multi-attribute utility scores where 1 represents perfect health, 0 represents death, and scores less than 0 are possible. The EQ-5D-5 L also includes a 0–100 VAS recording overall health status with higher scores representing better health.
- Doleur Neuropathique Questionnaire (DN4) [21]: seven yes/no questions with total scores being the number of questions answered yes. Scores of 3 or greater are considered indicative of neuropathic pain.
- Patient-reported scar assessment using the patient scale from the Patient and Observer Scar Assessment Scale (POSAS) [22]: six questions each scored out of 10. The answers are summed to give an overall score out of 60 with higher scores indicating better healing. This scale is also measured at 30 days post-injury.
- Complications grouped into three categories: (i) local complications related to the injury or operation—this will include wound healing assessment at 30 days using photographs, signs of infection up to 6 months, and other local complications; (ii) systemic complications related to the injury or operation—this will include other related SAEs; and (iii) unrelated SAEs.

### Sample size

Only one previous randomised trial has compared NPWT to standard dressings for surgical incisions associated with major trauma to the lower limb [12]. This trial indicated that the rate of ‘late’ (deep) infection was reduced by 6%; from 15% in the standard treatment group to 9% in the NPWT group.

In the absence of a minimum clinically important difference for deep wound infection, we surveyed surgeons in the UK Orthopaedic Trauma Society who perform surgery for major trauma to the limbs (unpublished data, 2015). This survey showed that a 6% reduction in the rate of ‘deep infection’ would, universally, be sufficient to change clinical practice with regard to the choice of dressing. Therefore, assuming a reduction in the proportion of patients having a deep infection from 15% to 9%, 615 patients would be required in each group to provide 90% power at the 5% level (two-sided) when comparing two independent proportions. Previous experience in clinical trials of lower limb fracture surgery for major trauma indicates that up to 20% of the primary outcome data may be lost during the follow-up period due to death and loss to follow-up [23]; therefore, we propose to recruit 1540 patients in total for this trial (770 per arm).

### Statistical analysis

#### General analysis principles

Two analysis populations will be considered, the intent-to-treat (ITT) population and the per-protocol (PP) population. The ITT population will include all participants randomised with the exception of those who: (i) prospectively declined consent but were subsequently randomised in error; (ii) retrospectively declined consent and requested that all their data were removed; or (iii) withdrew and requested that all their data were removed. Participants will be analysed according to the group to which they were randomised. The PP population will be analysed according to the treatment they actually received. Participants with major protocol deviations or violations will be excluded from the PP population. Major protocol deviations are those who did not satisfy the eligibility criteria (for example their wound could not be closed primarily), those who did not receive the allocated treatment, and those for whom insufficient data are available on the primary outcome. The definition of the PP population will be finalised during a blinded analysis of the data prior to the primary analysis time point.

A significance level of 0.05 will be used throughout, and 95% confidence intervals will be reported. The primary conclusion of the trial will be based on the results from the primary analysis of the primary outcome. Sensitivity analyses of the primary outcome will be performed to assess whether these results are robust. All analyses of secondary outcomes will be considered as supporting the primary analysis, and conclusions of the trial will not be based on these outcomes.

All analyses will be carried out using appropriate, validated statistical software such as STATA [24] or R [25]. The relevant package and version number used for the analysis will be recorded and reported.

#### Descriptive analyses

**Fig. 1:**
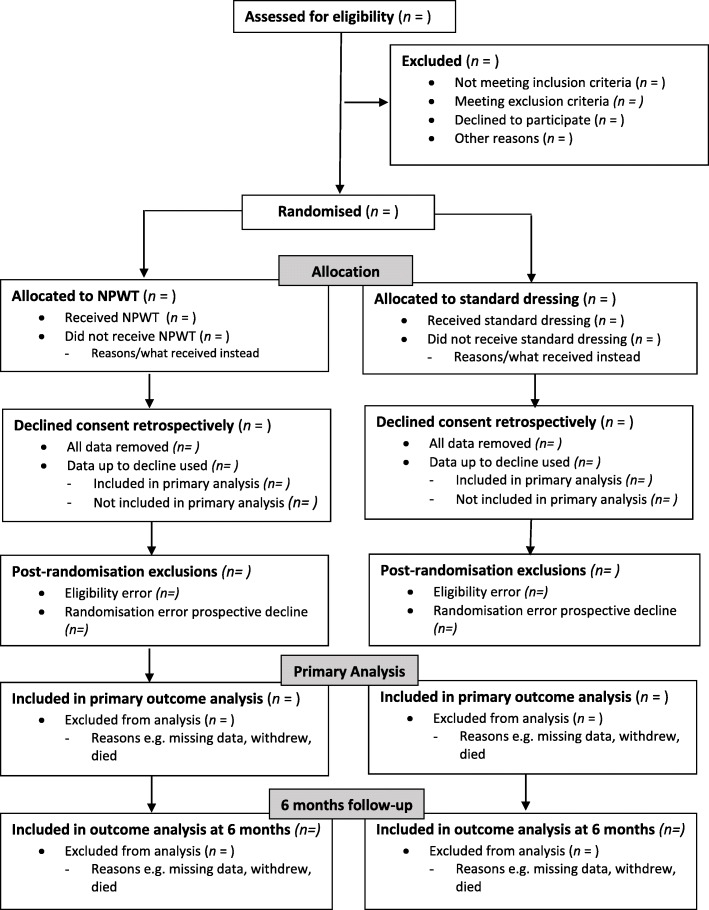
CONSORT flow diagram for participants in trial up to 6 months follow-up

The flow of participants through each stage of the trial, including the number of eligible individuals screened, randomised to each arm, receiving allocated treatment, and included in the primary analysis will be summarised using a CONSORT flow chart (Fig. 1). Reasons for ineligibility, loss to follow-up, and exclusion from the primary analysis will be summarised, as will the number of patients declining consent both prospectively and retrospectively.

The baseline comparability of the two randomised groups in terms of (i) minimisation factors, (ii) baseline characteristics (Table 1), (iii) operative procedure details (Table 2), and (iv) secondary outcomes at baseline will be presented. Numbers with percentages will be used to compare binary and categorical variables, and either means and standard deviations or medians and interquartile ranges will be used for continuous variables. There will be no tests of statistical significance nor confidence intervals for differences between the randomised groups.

**Table 1:** Baseline characteristics

Baseline characteristic	Type	Levels or scale
Sex	Binary	Male; female
Age	Continuous	Years
BMI	Continuous	kg/m^2^
Marital status	Categorical	Single; separated; married/civil partner; living with a partner; divorced; widowed
Ethnicity	Categorical	White; Black Caribbean; Black African; Black other; Indian; Pakistani; Bangladeshi; Chinese; other
Training post school	Categorical	None; formal work qualification; college/university non-degree; degree from college/university
Employment status	Categorical	Full-time employed; part-time employed; self-employed; retired/looking after home/inactive; unpaid work; unemployed; full-time student
Mechanism of injury	Categorical	Low energy fall; high energy fall; road traffic accident; crush injury; contact sports injury; other
Any other injuries	Binary	Yes; no
Diagnosed with diabetes	Binary	Yes; no
Regular smoker	Binary	Yes; no
Alcohol consumption per week	Categorical	0–7 units; 8–14 units; 15–21 units; more than 21 units
Regular analgesia before injury	Binary	Yes; no
Other medication before injury	Binary	Yes; no

**Table 2:** Operative procedure details

Operative procedure detail	Type	Level or scale
Lead surgeon grade	Categorical	Consultant; associate specialist; specialist trainee; other
Number of surgeons	Continuous	NA
Wound limb	Binary	Right; left
Wound location	Categorical	Hip; femur; patella; tibia; foot
How fixed	Categorical	Nail; plate and screws; wire/tension band wires; half-pin; fine pin; other
How closed	Categorical	Interrupted sutures; skin clips; subcuticular suture; any skin closure used; steristrips; glue; other
Intra-operative complications	Binary	Yes; no
If yes what	Categorical	Nerve injury; vascular injury; tendon injury; extension of fracture; other
Any other surgery	Binary	Yes; no
If yes what	Categorical	Head; chest; abdomen; pelvis; spine; upper limbs; ipsilateral limb; contralateral limb
Prophylactic antibiotics	Binary	Yes; no
Duration of operation	Continuous	Minutes

#### Loss to follow-up, withdrawals, and missing data

The numbers (and percentages) of losses to follow-up and withdrawals along with reasons for these will be reported by intervention arm at each time point. To ensure that differential losses do not occur between the two groups this will be tested using absolute risk differences (with 95% confidence intervals) and a chi-squared test. Any deaths and their causes will be reported separately.

The patterns of availability of data for primary and key secondary outcomes, from baseline to end of follow-up, will be summarised for the two treatment groups (as number and percentage of individuals missing). Reasons for missing-ness will also be presented, if known. Where appropriate, differentiation will be made between partially completed and fully missing outcome data. Two analysis datasets will be considered: (i) the available case dataset, consisting of all observed data; and (ii) the imputed dataset, where missing data are imputed. Missing data on the primary outcome will be imputed using a best case worst case analysis, considering the situation where all participants in the Standard dressing group with missing data are assumed to have a deep infection and all participants in the NPWT group with missing data are assumed not to have a deep infection and vice versa. Missing data on continuous outcomes will be imputed using multiple imputation (MI) under the missing at random (MAR) assumption. The suitability of the MAR assumption will be considered.

#### Compliance

The randomised intervention in this trial is the dressing (standard or NPWT) applied to the closed fracture wound at the end of surgery. This intervention occurs at a single time point, and compliance is therefore defined as the proportion of participants in each arm receiving the treatment to which they were randomised. The number (and percentage) of participants receiving the assigned dressing and receiving another dressing or no dressing in each arm will be summarised as well as the reasons for not receiving the randomised treatment. Details of what was provided instead will also be summarised.

#### Analysis of primary outcome

The numbers and percentages of ‘deep infections’ occurring up to 30 days post-randomisation in the two study intervention groups, NPWT and standard dressing, will be calculated. The rates of deep infection in the two study groups will be compared using a mixed effects logistic regression model. The model will include a random effect to account for any heterogeneity in the response due to recruitment centre. Fixed effects to adjust for open versus closed fractures, ISS level (≤ 15 vs ≥ 16), participant age, and participant gender will also be included. Participant age and gender are included in the model since there is evidence that older men have worse outcomes after major trauma. The result will be reported as an odds ratio (OR) with associated 95% confidence interval and *p* value for comparison between the two treatment groups. The unadjusted OR and associated 95% confidence interval will also be reported.

This analysis will be conducted for the ITT population using the available case dataset. As sensitivity analyses, the analysis will be repeated for: (i) the ITT population using an imputed dataset (best case worst case analysis); and (ii) the PP population using the available case dataset. In addition, a sensitivity analysis taking account of the competing risk of death [26] will be conducted if a sufficient number of deaths have occurred prior to 30 days, that is if more than 5% of participants have died prior to this time point.

If a significant treatment effect of NPWT is identified in the primary analysis, an exploratory subgroup analysis will be conducted to investigate whether this effect is moderated by the underlying risk level of the wound. This will be done by repeating the primary analysis and including wound location (above or below the knee) as a covariate. Wound location will be used as a proxy for wound risk level due to differences in soft-tissue cover; there being less soft-tissue cover below the knee.

The main analysis of the primary outcome (ITT population using the available case dataset) will be repeated using the alternative definition of ‘deep infection’, including infections occurring up to 90 days after injury. If any of the sensitivity analyses conducted for the primary endpoint (30 days) demonstrated substantially different results from the primary analysis, these analyses will be repeated for the rates of deep infection up to 90 days.

#### Analysis of secondary outcomes

For each of the continuous secondary outcomes (DRI, EQ-5D-5 L, and POSAS) the mean and standard deviation for each intervention arm will be reported. Assuming approximate normality is established, multi-level mixed-effects linear regression models, using repeated measures (level 1) nested within participants (level 2), will be used. The suitability of the assumption of approximate normality will be explored by plotting the residuals from this model. The model will include a random effect to account for any heterogeneity in response due to recruitment centre (level 3). The model will also include fixed effects to adjust for open versus closed fractures, ISS level (≤ 15 vs ≥ 16), participant age and participant gender, and, where appropriate, pre-injury values (DRI and EQ-5D-5 L). Trends over time in each intervention arm will be examined by plotting these, and, if trends differ between arms, interactions between treatment and time will be included in the model. The adjusted difference between the treatment arms at each time point will be reported. This analysis will be conducted for the ITT population using the available case dataset. The analysis of the DRI will be repeated using an imputed dataset. Data will be imputed using MI under the MAR assumption.

If, for any of these variables, approximate normality is not appropriate, the first approach will be to consider a transformation of the data or the use of a different metric such as change from baseline to attain normality. If normality cannot be achieved by transformation, the data will be analysed using a non-parametric equivalent (Mann-Whitney U-test) with no adjustment and medians and interquartile ranges will be reported for each treatment arm.

In addition, supplementary analyses of the DRI and EQ-5D utility variables will be conducted using area under the curve (AUC) summary statistics [27]. For each intervention arm, a linear combination of the parameter estimates at each time point (baseline to 6 months) from the mixed effects models will be used to calculate the AUC, thus providing an overall estimate of recovery over time for each intervention arm. This analysis will be conducted for the ITT population using the available cases dataset. The difference between the two groups will be calculated and compared using a *t*-test.

The DN4 will be analysed using similar methods to those outlined for the primary outcome. The number and proportion of individuals deemed to have neuropathic pain (DN4 ≥ 3) will be reported for each treatment arm. A multi-level mixed-effects logistic regression model with repeated measures (level 1) nested within participants (level 2) will be used. The model will be adjusted for recruitment centre as a random effect (level 3), and fixed effects will be included to adjust for open versus closed fractures, ISS level (≤ 15 vs ≥ 16), participant age, and participant gender. Trends over time will be examined, and, if appropriate, interactions between treatment and time will be included. Results will be presented as ORs with associated confidence intervals. The unadjusted OR and associated 95% confidence interval will also be reported. This analysis will be conducted for the ITT population using the available case dataset.

Similar methods will also be used to analyse complications. The number and percentage of people experiencing each complication in each treatment arm will be reported. If there are sufficient numbers of events, a mixed-effects logistic regression model will be used to compare the rates of complications between intervention arms. The model will include a random effect for recruitment centre and fixed effects for open versus closed fractures, ISS level (≤ 15 vs ≥ 16), participant age, and participant gender. If there are not sufficient numbers of events to fit an adjusted model, unadjusted differences between intervention arms will be calculated using a chi-squared test. This analysis will be conducted for the ITT population using the available case dataset. Temporal patterns of complications will be presented graphically and, if appropriate, a time-to-event analysis (Kaplan-Meier survival analysis) will be used to assess the overall risk and risk within individual classes of complications.

## Discussion

The WHIST trial will initially provide data regarding the effects of NPWT on the outcomes of participants up to 6 months after surgery for major trauma, compared to those receiving standard wound dressings. This paper provides details of the planned statistical analyses for this trial and will help reduce the risks of outcome reporting bias and data-driven results [28]. The participants enrolled in the trial will subsequently be followed up annually for 5 years to assess their long-term outcomes, but the analysis of these data will be reported separately.

## Trial status

Recruitment for the trial closed on 17 April 2018. In total 1548 patients from 24 study sites were recruited. Follow-up is currently ongoing and expected to finish in October 2018; the analysis of outcomes up to 6 months after randomisation will be conducted thereafter. The trial also includes long-term follow-up from 1 to 5 years and the analysis of these data will be reported separately.

## Abbreviations

AUC: Area under the curve
CDC: Centre for Disease Control and Prevention
DN4: Doleur Neuropathique Questionnaire
DRI: Disability Rating Index
ISS: Injury Severity Score
ITT: Intent-to-treat
MAR: Missing at random
MI: Multiple imputation
NPWT: Negative-pressure wound therapy
OR: Odds ratio
POSAS: Patient and Observer Scar Assessment Scale
PP: Per-protocol
SAE: Serious adverse event
TARN: Trauma Audit and Research Network
VAS: Visual analogue scale
WHIST: Wound Healing In Surgery for major Trauma

## References

[1] National Institute for Health and Care Excellence. Major trauma: Service delivery (NICE Guideline 40). 2016. Available at: https://www.nice.org.uk/guidance/ng40/evidence/full-guideline-pdf-2313258877. Accessed 22 Mar 2019. 26913313

[2] Stokel EA, Sadasivan KK (1991). Tibial plateau fractures: standardized evaluation of operative results. Orthopedics.

[3] Stannard J, Martin S, Stannard J, Schmidt A, Kregor P (2007). Tibial plateau fractures. Surgical treatment of orthopaedic trauma. edn.

[4] Mallik A, Covall D, Whitelaw G (1992). Internal versus external fixation of bicondylar tibial plateau fractures. Orthop Rev.

[5] Koval KJ, Helfet DL (1995). Tibial plateau fractures: evaluation and treatment. J Am Acad Orthop Surg.

[6] Young M, Barrack R (1994). Complications of internal fixation of tibial plateau fractures. Orthop Rev.

[7] Wyrsch B, Mcferran MA, Mcandrew M, Limbird TJ, Harper MC, Johnson KD, Schwartz HS (1996). Operative treatment of fractures of the tibial plafond. A randomized, prospective study. JBJS.

[8] Teeny SM, Wiss DA (1993). Open reduction and internal fixation of tibial plafond fractures: Variables contributing to poor results and complications. Clin Orthop Relat Res.

[9] McFerran MA, Smith SW, Boulas HJ, Schwartz HS (1992). Complications encountered in the treatment of pilon fractures. J Orthop Trauma.

[10] Blauth M, Bastian L, Krettek C, Knop C, Evans S (2001). Surgical options for the treatment of severe tibial pilon fractures: a study of three techniques. J Orthop Trauma.

[11] MacKenzie EJ, Castillo RC, Jones AS, Bosse MJ, Kellam JF, Pollak AN, Webb LX, Swiontkowski MF, Smith DG, Sanders RW (2007). Health-care costs associated with amputation or reconstruction of a limb-threatening injury. JBJS.

[12] Stannard JP, Volgas DA, McGwin G, Stewart RL, Obremskey W, Moore T, Anglen JO (2012). Incisional negative pressure wound therapy after high-risk lower extremity fractures. J Orthop Trauma.

[13] Webster J, Scuffham P, Stankiewicz M, Chaboyer WP (2014). Negative pressure wound therapy for skin grafts and surgical wounds healing by primary intention. Cochrane Database Syst Rev..

[14] Achten J, Vadher K, Bruce J, Nanchahal J, Spoors L, Masters JP, Dutton S, Madan J, Costa ML (2018). Standard wound management versus negative-pressure wound therapy in the treatment of adult patients having surgical incisions for major trauma to the lower limb—a two-arm parallel group superiority randomised controlled trial: protocol for Wound Healing in Surgery for Trauma (WHIST). BMJ Open.

[15] Gamble C, Krishan A, Stocken D, Lewis S, Juszczak E, Doré C, Williamson PR, Altman DG, Montgomery A, Lim P (2017). Guidelines for the content of statistical analysis plans in clinical trials. JAMA.

[16] Horan TC, Andrus M, Dudeck MA (2008). CDC/NHSN surveillance definition of health care–associated infection and criteria for specific types of infections in the acute care setting. Am J Infect Control.

[17] National Healthcare Safety Network, Centers for Disease Control and Prevention. Surgical site infection (SSI) event. https://www.cdc.gov/nhsn/pdfs/pscmanual/9pscssicurrent.pdf. Accessed 20 Feb 2018.

[18] Salen BA, Nordemar R (1994). The Disability Rating Index: an instrument for the assessment of disability in clinical settings. J Clin Epidemiol.

[19] Brooks R (1996). EuroQol: the current state of play. Health Policy.

[20] Herdman M, Gudex C, Lloyd A, Janssen M, Kind P, Parkin D, Bonsel G, Badia X (2011). Development and preliminary testing of the new five-level version of EQ-5D (EQ-5D-5L). Qual Life Res.

[21] Bouhassira D, Lantéri-Minet M, Attal N, Laurent B, Touboul C (2008). Prevalence of chronic pain with neuropathic characteristics in the general population. Pain.

[22] Draaijers LJ, Tempelman FR, Botman YA, Tuinebreijer WE, Middelkoop E, Kreis RW, van Zuijlen PP (2004). The patient and observer scar assessment scale: a reliable and feasible tool for scar evaluation. Plast Reconstr Surg.

[23] Costa ML, Achten J, Griffin J, Petrou S, Pallister I, Lamb SE, Parsons NR (2017). Effect of locking plate fixation vs intramedullary nail fixation on 6-month disability among adults with displaced fracture of the distal tibia: The UK FixDT randomized clinical trial. JAMA.

[24] StataCorp (2017). Stata Statistcial Software: Release 15.

[25] R Core Team (2017). R: A language and environment for statistical computing.

[26] Varadhan R, Weiss CO, Segal JB, Wu AW, Scharfstein D, Boyd C (2010). Evaluating health outcomes in the presence of competing risks: a review of statistical methods and clinical applications. Med Care.

[27] Bell ML, King MT, Fairclough DL (2014). Bias in area under the curve for longitudinal clinical trials with missing patient reported outcome data: summary measures versus summary statistics. SAGE Open.

[28] Finfer S, Bellomo R (2009). Why publish statistical analysis plans. Crit Care Resusc.

